# Characteristics of Gun Advertisements on Social Media: Systematic Search and Content Analysis of Twitter and YouTube Posts

**DOI:** 10.2196/15736

**Published:** 2020-03-27

**Authors:** Lisa Jordan, James Kalin, Colleen Dabrowski

**Affiliations:** 1 Drew University Madison, NJ United States

**Keywords:** firearms, advertising, social media, internet, gender identity

## Abstract

**Background:**

Although gun violence has been identified as a major public health concern, the scope and significance of internet gun advertising is not known.

**Objective:**

This study aimed to quantify the characteristics of gun advertising on social media and to compare the reach of posts by manufacturers with those of influencers.

**Methods:**

Using a systematic search, we created a database of recent and popular Twitter and YouTube posts made public by major firearm manufacturers and influencers. From our sample of social media posts, we reviewed the content of the posts on the basis of 19 different characteristics, such as type of gun, presence of women, and military or police references. Our content analysis summarized statistical differences in the information conveyed in posts to compare advertising approaches across social media platforms.

**Results:**

Sample posts revealed that firearm manufacturers use social media to attract audiences to websites that sell firearms: 14.1% (131/928; ±2.9) of Twitter posts, 53.6% (228/425; ±6.2) of YouTube videos, and 89.5% (214/239; ±5.1) of YouTube influencer videos link to websites that facilitate sales. Advertisements included women in efforts to market handguns and pistols for the purpose of protection: videos with women included protection themes 2.5 times more often than videos without women. Top manufacturers of domestic firearms received 98 million channel views, compared with 6.1 billion channel views received by the top 12 YouTube influencers.

**Conclusions:**

Firearm companies use social media as an advertising platform to connect viewers to websites that sell guns. Gun manufacturers appropriate YouTube servers, video streaming services, and the work of YouTube influencers to reach large audiences to promote the widespread sale of consumer firearms. YouTube and Twitter subsidize gun advertising by offering server and streaming services at no cost to gun manufacturers, to the commercial benefit of Google and Twitter’s corporate ownership.

## Introduction

### Background

Gun production and imports of guns in the United States have risen significantly over the past 30 years. According to the Bureau of Alcohol, Tobacco, Firearms, and Explosives, there has been a three-fold increase in the total number of guns made in the United States from 3 million in 1986 to over 11 million in 2016. Imports grew from 0.7 million in 1986 to 4.49 million in 2017 [1]. As the international regulation of firearms in most industrialized countries tends to be more restrictive than the United States, some foreign manufacturers, such as those in Japan, export over 90% of their annual production to the United States [2,3]. Proliferation of firearms presents significant challenges to domestic and global public health: weapons from US markets have been linked to elevated crime, violence, and homicide in other countries [3-8]. Public health responses to firearms require a closer examination of the company practices that facilitate the widespread distribution and increased lethality of small arms in the United States.

US gun control research has much to learn from successful public health programs that sought to reduce widespread injuries from motor vehicle accidents and harm from tobacco use. For example, in outlining his recommendations for a public health approach to guns, Hemenway [3] borrowed the Haddon matrix [9] from injury prevention studies to illustrate opportunities for health interventions before, during, and after injury events involving firearms. Hemenway also recommended that public health scholarship examine the approaches that firearm manufacturers use to promote gun sales, drawing comparisons to very successful health interventions in tobacco advertising and sales [3]. Interventions aimed at restricting the distribution of firearms, limiting gun advertising, and challenging the normalization of gun use are examples of prevention programs that reduce firearm-related injuries.

Although several past studies describe the characteristics of firearm advertising in print magazines and catalogs [2,10,11], the landscape for advertising, sales, and communications has changed radically with the advent of Web-based marketing. New public health scholarship in this area is needed [12,13]. However, despite diversification in the media to include Web-based videos, websites, blogs, podcasts, and social media, many of the advertising messages developed over the past few decades are similar to current approaches, making previous research still relevant for informing contemporary studies of social media and Web-based gun sales.

Building on work to characterize print advertising [2,10,14], this paper examined major themes in firearm advertising used in social media. First, we developed a system for sampling and comparing Twitter use by firearm manufacturers to distribute advertising, then we expanded our work to classify YouTube posts made by gun manufacturers and gun influencers to study firearm advertising. We have begun with a descriptive review of US civilian firearm ownership, the health consequences of firearm advertising, and the role of social media in advertising. Our methods for studying the advertising characteristics used by firearm manufacturers have been described, and the descriptive results of our analysis have been provided. Finally, we concluded with a list of possible interventions to curtail small arms proliferation in US civilian markets and a list of recommendations for future work.

### Characteristics of Civilian Gun Ownership in the United States

Recent estimates of US civilian firearms place the total number of private guns at 393 million: higher than the US population [15]. Despite the high overall volume of weapons, the General Social Survey and Pew Research Surveys found a trend toward declining US gun ownership, from over half of adults in 1980 to less than one-third of adults in 2015 [16]. Concurrent with the decline in ownership is a rise in the number of guns per owner: the National Firearms Survey estimated that half the US civilian gun stock was owned by 14% of the gun owners, comprising roughly 3% of the US population [17].

Trends in US gun ownership are stratified by time, place, and demography. At no time in US history has ownership of this lethal weapon been equal. The General Social Survey results reveal significant regional and temporal variations in ownership over the past 50 years. The highest ownership rates were found in 1976, where over 80% of households located in East South Central states (Alabama, Kentucky, Mississippi, and Tennessee) reported owning a gun; conversely, in 2018, sample data for Middle Atlantic states (New York, New Jersey, and Pennsylvania) estimate ownership rates at 19% [18]. Contemporary studies found that 67% of gun owners respond that protection is a major reason for owning a gun [19].

The Pew Research Center found substantive demographic differences in US gun ownership: 39% of men and 22% of women recently surveyed own a gun [20]. White gun ownership is 50% greater than black gun ownership (36% compared with 24%), whereas Hispanic gun ownership is less than half the rate of white gun ownership (Hispanic gun ownership is estimated at 15%) [20]. One study found that public perception of gun ownership significantly overestimates actual ownership, which may contribute to more moderate views of gun control [21]. To reiterate, the majority of people in the United States do not own a gun, but male and white populations own guns at significantly higher rates.

### Health Outcomes and Gun Advertising

The health impact of firearms in the United States is widespread. In total, 44% of US residents know someone who has been shot, and a higher proportion, 51% of US gun owners surveyed, know someone who was shot [22]. When combined across causes of death, including firearm deaths from homicides, suicides, and accidental shootings, the annual age-adjusted death rate declined from a peak in 1993, but the total number of firearm-related deaths in the United States has increased in recent years and in 2017 it reached 39,773, exceeding total deaths from motor vehicle accidents [22,23]. According to the Centers for Disease Control, from 1999 to 2017, 612,000 deaths by firearms occurred in the United States [23].

Many scholars have observed the significance of firearms on children’s health: gun-related injuries are the second leading cause of death for children and adolescents in the United States [24]. Over 70% of gun owners have small children, and firearm-related deaths are more frequent when handgun ownership is higher [25]. A study of school-associated homicides in the United States found that although rates of single-victim homicides remained unchanged over the past 20 years, multiple-victim incidence rates increased significantly from 2009 to 2018 [26]. In total, 95% of multiple-victim school-associated homicides in the United States were from firearm-related injuries, compared with the rate of 62.8% for single-victim homicides [26].

The presence of firearms is a known hazard. Occupations that require firearm use have been connected to elevated risk of fatality by suicide. Data from the northeastern United States found that 13% of suicide attempts resulted in fatality; however, 91% of suicide attempts by firearms were lethal [27]. Suicides among US police officers, veterans, and members of the armed forces have come under particular scrutiny. Recent studies have shown that the rate of suicide among veterans and service members is twice the rate of suicide in the civilian population and that firearms were used in 70% of service member suicide deaths [28]. New York Police Department suicides have been declared a mental health emergency [29]. Evidence-based interventions in the Israeli military that restricted officer access to firearms over the weekend reduced suicide deaths by 40% [30].

The risk of firearm injury found in occupational health extends to the general population. Research in preventive medicine found US gun ownership and youth suicide rates to be closely correlated [31] and that state legal restrictions on firearms reduced intimate partner homicide [32]. Permissive state gun laws are also significantly associated with greater numbers of mass shootings [33].

Perhaps in response to the high burden of deaths from firearms, 67% of Americans surveyed by the Associated Press in partnership with the University of Chicago in 2017 and 2018 favor stricter gun laws [34]. Community-based interventions that provide gun storage for families with suicidal individuals may also save lives, suggesting that where fewer state laws exist, law enforcement and gun dealers may step in to promote safe storage [35]. A recent survey of gun owners identified law enforcement and active military as the most effective groups to educate about safe gun storage [36].

Some scholars equate advertising with disease promotion. Freudenberg [37] argued:

Advertising seeks to create new customers and encourage existing ones to purchase more. When the product being advertised is lethal (as in the case of tobacco or guns) or can easily be used in ways that harm health (e.g. alcohol, SUVs, and some pharmaceuticals), advertising falls squarely within the rubric of disease promotion.

Both the expansion of US civilian gun markets and the intensification of gun ownership to include more weapons are troubling trends, given recent evidence that individuals in the United States who self-report *impulsive angry behavior* and gun ownership currently comprise an estimated 8.9% of the population [38]. Reducing the appeal of assault weapons, and limiting advertisements of guns to police, veterans, and armed service members, by better understanding and intervening in gun advertising could translate into fewer gun-related deaths.

### Gun Advertising on Social Media

Decisions by major print, television, and Web-based media sources to restrict paid firearm advertising is remarkable: gun advertisements used to circulate among major US newspapers and magazines commonly found in homes [11]. Notable advocacy campaigns, such as *Close the Loophole on Gun Advertising*, went from newspaper to newspaper to negotiate policies that would limit gun solicitations in classified advertisements [37]. Comcast followed NBC, Time Warner Cable, Fox, and ESPN to ban firearm and ammunition advertising in most channels in 2013 [39].

Despite encouraging statements by private media companies, public health responses to predatory advertising have been limited by the 2005 passage of the federal Protection of Lawful Commerce in Arms Act (PLCAA), which protects gun makers from lawsuits related to weapons misuse [14,40]. Although firearm advertisers have acknowledged constraints, such as exclusions from Super Bowl advertising, they have welcomed looser, more diversified Web-based options through both mainstream and alternative internet and social media [41].

The shift to internet advertising has been rapid. Since 2005, the number of US adults who used a social media site grew from 5% to 69% in 2018 [42]. The number is highest among adults aged between 18 and 29 years: 88% [42]. In 2019, the most commonly used social media platform by US adults was YouTube (73%) followed by Facebook (69%) and Twitter (22%) [43]. Among adults aged between 18 and 29 years, over 90% said they have used YouTube, and 44% of adults aged 18 to 24 years said they have used Twitter [43]. According to YouTube, 1.9 billion users log on each month, from over 90 countries [44].

Most US adult YouTube users agree that the site is very important for “figuring out how to do things they haven’t done before” [45]. However, users often identify problematic content. Among US adults viewing YouTube, 61% say they frequently or sometimes have observed videos with “people engaging in dangerous or troubling behavior” [45]. Moreover, 81% of US parents let their children watch videos on YouTube, and 61% of those parents have felt that their child regularly or occasionally “encountered content that they felt was unsuitable for children” [45].

The relative efficacy of social media advertising leading to firearm sales is unknown: most manufacturers are privately owned companies, so documents on advertising expenditures and company profitability are not public [2,3]. However, there is an emerging, but rich, body of research on the use of social media, particularly YouTube, for spreading tobacco and e-cigarette advertising [46-51]. Given there are restrictions on legal channels for advertising, tobacco companies have increasingly turned to internet promotion [52]. Platforms such as YouTube present challenges for consumer information, because it is difficult to differentiate paid advertising from purely creative content, and the authenticity of YouTube videos creates relationships between the video personalities and the viewers, which are particularly influential with young people [53]. In the case of tobacco, exposure to Web-based marketing was found to be a risk factor for use [54-56].

Celebrity endorsements have been found to positively influence sales, and previous research identifies Twitter and other social media platforms as important mechanisms to share endorsements [57]. In addition to celebrities, an increasingly scrutinized source of behavioral change is the role of the internet or social media influencers. For example, recent studies connected tobacco use behaviors to influencers on Twitter [58], and research on eating behaviors in youths found that YouTube influencers significantly affected unhealthy food consumption [59]. Studies now connect *engagement* strategies with increased advertising effectiveness [55,60]. Research also found that tobacco engagement marketing led to an increase in “the risk of initiation and progression and decrease in likelihood of cessation” [55].

Pew Research Center surveys reveal some of the patterns in media consumption among gun owners: 43% of male gun owners (33% of female gun owners) watch television programs or videos about guns, and 39% of male gun owners (28% of female gun owners) visit websites about guns [20]. Researchers also found that gun owners with more guns were more likely to watch gun videos or visit gun websites: 53% of gun owners with 5 or more firearms watch videos about guns, compared with 32% of gun owners with only 1 gun [20]. In total, 51% of gun owners with five or more guns visited gun websites, compared with 22% who owned only 1 gun [20].

Protection is a dominant concern for gun owners and a common marketing message used by manufacturers of guns. According to Pew Research Center findings, two-thirds of gun owners say that protection is the major reason that they own a gun [22]. Handgun advertising has been shown to exploit this rationale, despite evidence that households with firearms are at elevated risks of gun violence from homicide, suicide, and accidental injury [61].

Social networks also appear to influence a variety of health behaviors [62]. *Screenagers*, a documentary by Delaney Ruston, a primary care physician, describes the wide range of negative health and behavioral outcomes emerging from social media use by the youth [63]. Adoption of social media to create discord and harm poses a threat to public health: one study has explored violence and crime-related Twitter use by gangs in Detroit, Michigan [64]. Another recent article found troubling evidence for the use of Russian bots to distribute misinformation about vaccines on Twitter [65].

In some ways, internet and social media companies have been responsive to gun violence and other issues. Social media companies agreed to a variety of restrictions after the Parkland school shooting [66]. YouTube expanded its previous ban on videos demonstrating the use or construction of bump stock modifications following the Las Vegas shooting in 2016 to include more rigorous restrictions [67]. According to stated policy, videos intending to sell firearms, providing instruction on the construction of firearms, ammunition, or accessories, or providing instruction on the installation of accessories are not allowed and will result in the removal of the video [68]. However, the details of the policy description focus on person-to-person sales and do-it-yourself fabrication as the emphasis of content moderation. As of June 2019, Twitter’s policy is as follows: “Twitter prohibits the promotion of weapons and weapon accessories globally” [69]. Nevertheless, many are concerned that protections for viewers, particularly for children, are too relaxed and that implementation of company policies has been less effective than socially desired [70].

Recent research on commercial content moderation challenges the face value of social media policies, demonstrating the intentionality behind vague user guidelines and the purposeful cover-up of company instruments of control [71,72]. For example, most commercial content moderation requires human intervention that is not automated [71,72]. The employment and working conditions, particularly with respect to occupational health and mental health, are shocking, unethical, and hidden behind restrictive nondisclosure agreements or by recruiting work through contract labor, piecemeal labor, and offshore arrangements [72].

This study aimed to characterize the contemporary use of social media for the purposes of gun advertising by gun companies and YouTube influencers. We quantified the frequency of common themes found in gun advertisements, as first designated by research pertaining to print advertisements [10]. This study builds on past research of print advertisements by conducting a systematic search of Twitter and YouTube use by major US gun manufacturers, identifying links to internet gun sales made in Twitter and YouTube posts, and conducting a systematic search of gun promotion found in YouTube influencer posts.

## Methods

To study the ways that firearm manufacturers use social media for advertising, we performed a systematic search and content analysis. We began by identifying top gun manufacturers. Next, we located the publicly accessible Twitter profiles and YouTube channels connected to the manufacturing companies. We sampled the manufacturer posts by examining the most recent and most popular posts. From our sample, we systematically reviewed the text, images, and video information delivered on each post for the presence of 19 advertising themes, originally explored in a previous study by Saylor et al [10]. These variables were coded into two databases: Twitter posts and YouTube posts.

In addition to surveying information distributed by firearm manufacturers on social media, we chose to explore the recent and most popular posts made by YouTube influencers. On the basis of marketing reports from the firearm advertising industry, we searched for the most influential YouTube channels that focus on sharing information about firearms. This sample of social media posts was also reviewed for the presence of gun advertising themes. After the observations of social media posts were complete, we conducted a content analysis by calculating summary statistics to describe the characteristics of posts made by manufacturers on Twitter and YouTube and by influencers on YouTube. We compared the recent posts with the most popular posts, across platforms and owners.

The specific process for identifying social media advertisements is outlined below. We began with a systematic search of top domestic gun producers and identified top foreign imports to the United States. We explored the relative and cumulative impact of their production of guns, and their relative and cumulative impact on social media advertising. We also specified the database design and contents.

Producers of firearms and ammunition in the United States and the quantities of guns made annually are listed in the *Annual Firearms Manufacturing and Export Report*, published by the Bureau of Alcohol, Firearms, Tobacco, and Explosives (ATF; see Table 1 for a summary) [73]. Information aggregated by the ATF demonstrates that production of firearms is concentrated in a few companies. The top domestic manufacturer, Sturm, Ruger & Company, produced 1.6 million firearms in 2017, which comprised 19.50% (1,631,554/8,366,943) of all new domestic firearms. The top 4 companies manufacture the majority of all firearms (4,521,925/8,366,942, 54.04%), and the top manufacturers producing over 50,000 firearms in 2017, included 23 companies, accounting for 87.78% (7,345,049/8,366,942) of US production. Of the 2111 firearm manufacturers identified by the ATF, over half (n=1120) manufactured fewer than 10 firearms.

**Table 1:** Firearm manufacturing characteristics of top domestic producers (over 50,000 firearms produced in 2017).

Federal firearms licensed manufacturers	Types of firearms produced		Number of firearms manufactured (includes rifles, pistols, shotguns, revolvers, and miscellaneous), n (%)
	Rifles, n (%)	Pistols, n (%)	
Sturm, Ruger & Company	661,155 (40.52)	781,623 (47.91)	1,631,554 (19.50)
Smith & Wesson Corp	265,356 (17.62)	1,032,450 (68.54)	1,506,256 (18.00)
Remington Arms	448,513 (55.28)	59,581 (7.34)	811,421 (9.70)
Sig Sauer Inc	35,920 (6.27)	536,774 (93.73)	572,694 (6.84)
Maverick Arms Inc	80,275 (16.08)	0 (0.00)	499,100 (5.97)
Henry Rac Holding Corp	235,037 (100.00)	0 (0.00)	235,037 (2.81)
Heritage Manufacturing	0 (0.00)	0 (0.00)	226,065 (2.70)
Kimber Mfg Inc	11,378 (5.25)	183,858 (84.89)	216,585 (2.59)
WM C Anderson Inc	2295 (1.07)	1448 (0.67)	215,125 (2.57)
Glock Inc	0 (0.00)	175,696 (100.00)	175,696 (2.10)
Palmetto State Armory	28,562 (17.80)	3326 (2.07)	160,417 (1.92)
Springfield Inc	69,352 (46.01)	81,377 (53.99)	150,729 (1.80)
SCCY Industries LLC	0 (0.00)	150,647 (100.00)	150,647 (1.80)
Kel Tec CNC Industries	66,235 (43.97)	58,982 (39.16)	150,630 (1.80)
Radical Firearms LLC	88,430 (96.96)	2775 (3.04)	91,205 (1.09)
Strassells Machine Inc	40,511 (46.82)	46,015 (53.18)	86,526 (1.03)
Aero Precision LLC	1490 (1.87)	2 (0.00)	79,525 (0.95)
Beretta USA Corp	2778 (3.60)	57,411 (74.35)	77,214 (0.92)
FN America LLC	15,614 (20.25)	61,510 (79.75)	77,124 (0.92)
Taurus International	103 (0.15)	69,123 (100.00)	69,226 (0.83)
Colt's Manufacturing	13,942 (23.40)	31,987 (53.68)	59,591 (0.71)
Browning Arms	668 (1.30)	50,331 (97.82)	51,452 (0.61)
Diamondback Firearms	26,960 (52.63)	24,270 (47.37)	51,230 (0.61)
Total among top manufacturers (listed)	2094,574 (28.52)	3,409,186 (46.41)	7,345,049 (87.79)

For this paper, we chose to explore the top manufacturers that produced over 50,000 firearms. Given that a significant portion of firearms enter the United States as imports [2,74], and are not included in the domestic manufacturing list, we identified top manufacturers in other countries with sizable exports of firearms to the United States: Croatia (HS Produkt), Turkey (MKE, also known as Zenith Firearms), the Czech Republic (CZ firearms), and the Philippines (Armscor). Within the list of major producers, a few did not have Twitter handles (Sturm, Ruger & Company; Colt’s Manufacturing Company; and HS Produkt), and some did not have YouTube channels (Heritage Manufacturing; HS Produkt; and Radical Firearms). The SCCY Network YouTube channel was excluded because it only included two videos.

By searching Twitter, we were able to identify Twitter handles for 24 manufacturers, representing 68% of the domestic firearm production (Table 2). From these, we archived the 44,571 most recent tweets, ending May 15, 2019, representing 65% of all tweets from these manufacturers, using Twitonomy, a social media analytics service [75]. From the recent tweets obtained, we explored the 20 most recent tweets, ending May 15, 2019, and the 20 most retweeted tweets, for each company: generating a sample size of 928 tweets. The choice to explore 20 was arbitrarily made by the authors to create a sufficiently large sample to code within reasonable time constraints.

By searching YouTube, we found 24 channels hosted by the major firearm manufacturers, which account for 85% of the domestic firearm production (Table 3). Of the over 3600 videos posted by these manufacturers, we chose to classify the 10 most recent videos and the 10 most viewed videos. The number 10 was chosen arbitrarily by the authors. Our sample totaled to 425 videos, ranging in dates from May 2008 to May 2019. Overall, these channels covered over 0.5 million subscribers and 98 million views. The videos we reviewed account for 11.6% of all videos from these channels, but a sizable proportion of all views, at just over 44 million views.

**Table 2:** Twitter archive summary for top domestic firearm manufacturers by followers.

Manufacturer	Twitter handle	Total number of tweets, n	Followers, n
Glock Inc	@GLOCKInc	4954	290,240
Smith & Wesson Corp	@SmithWessonCorp	2844	265,906
Remington Arms Company LLC	@RemingtonArms	4924	240,378
Beretta USA Corp	@Beretta_USA	14,831	189,945
Springfield Inc	@Springfield_Inc	1634	144,283
Sig Sauer Inc	@sigsauerinc	2356	108,388
Kimber Mfg Inc	@kimberamerica	7646	75,738
Fn America, LLC	@FN_America	1695	68,417
CZ	@czusafirearms	3493	50,328
Aero Precision LLC	@aero_precision	691	43,939
Maverick Arms, INC (subsidiary of Mossberg & Sons)	@MossbergCorp	3480	40,109
Taurus International Manufacturing Inc	@TarususUSA	1785	23,941
Henry RAC Holding Corp	@HenryRifles	1251	17,227
Browning Arms Company	@BrowningArms	766	16,297
WM C Anderson Inc	@andersonrifles	6290	11,434
Sccy Industries LLC	@SCCYguns	1244	11,175
Kel Tec CNC Industries Inc	@keltecweapons	1330	9645
Palmetto State Armory, LLC	@PalmettoArmory	3891	9318
Diamondback Firearms LLC (owned by Taurus)	@DBFirearms	1048	5118
Armscor	@ArmscorRIA	571	3134
Strassells Machine Inc (also known as Hi-Point Firearms)	@HiPointFirearms	489	1559
Radical Firearms LLC	@RadicalFirearms	97	1112
MKE (also known as Zenith Firearms)	@ZenithFirearms	922	560
Heritage Manufacturing Inc	@heritagemfginc	473	131
Total	N/A^a^	68,705	1,628,322

^a^Not applicable.

**Table 3:** Summary of top firearm manufacturers on YouTube by total views.

Manufacturer	Subscribers, n	Total number of videos, n	Total number of views. n
SCCY Firearms, Sccy Industries LLC	Private	2	Private
Springfield Armory, Springfield Inc	Private	349	Private
Beretta USA Corp, Browning Arms Company	65,407	372	17,553,843
Sturm, Ruger and Co	74,282	364	15,133,053
Browning	18,532	617	13,191,067
Sig Sauer, Sig Sauer Inc	95,927	256	9,894,623
Smith and Wesson, Smith & Wesson Corp	43,865	233	8,696,129
Remington Arms, Remington Arms Company LLC	33,756	242	6,858,982
Mossberg, Maverick Arms, Inc (subsidiary of Mossberg & Sons)	27,593	115	5,631,875
Glock, Glock Inc	56,865	78	4,863,570
CZUSA, CZ	16,781	106	4,263,317
Taurus USA, Taurus International Manufacturing Inc	12,628	34	3,159,038
ArmscorRIA, Armscor	19,039	366	2,988,741
Henry Repeating Arms, Henry RAC Holding Corp	33,783	54	2,289,310
Kimber Firearms, Kimber Mfg Inc	12,418	188	1,489,968
Palmetto State Armory, Palmetto State Armory, LLC	17,190	57	870,149
Colt Manufacturing Co	7031	42	440,015
FN, Fn America, LLC	10,386	32	208,947
Aero Precision, Aero Precision LLC	9617	18	175,656
Zenith Firearms, MKE (also known as Zenith Firearms)	1528	50	146,328
Kel-Tec, Kel Tec CNC Industries Inc	441	41	61,618
Hi-Point, Strassells Machine Inc (also known as Hi-Point Firearms)	1,433	8	54,250
Anderson Manufacturing, WM C Anderson Inc	2,025	20	41,862
Diamondback Firearms, Diamondback Firearms LLC (owned by Taurus)	627	13	32,603
Total	561,154	3657	98,044,944

Firearm advertisers and advertisements refer to the important role of *influencers* in communicating information about new firearms and ammunition and promoting gun ownership and use for recreation and home protection. According to the Danger Close Media (DCM) Group, a firearm advertising organization, influencers are essential to firearm advertisers as social media outlets increase restrictions [76]. From the DCM Group, a list of top influencers was identified. We explored influencer channels on YouTube and Social Blade, a YouTube analytics website, to identify influencers that were not recognized by the DCM Group. Among over 4 dozen influencers found, we inspected the top 12 influencers, with over 150 million channel views. The list of influencers, along with channel names, views, subscribers, and short summaries can be found in Table 4. The top 10 and most recent 10 videos were reviewed for each channel based on the same attributes explored for Twitter and YouTube posts shared by manufacturers. The sample of influencers included a total of 239 videos.

Drawing from the methodology and categories originally identified by Saylor et al [10], which explored themes in firearm advertisements commonly appearing in print media, we reviewed our samples of 928 tweets, 425 videos by manufacturers, and 239 videos by influencers, across 19 characteristics, described in Table 5. We expanded our classification system for influencers to identify various forms of paid promotions. All characteristics listed in Table 5 were coded as binary variables.

Tweets by manufacturers typically included photos. Tweets often include video content, so Twitter and YouTube samples overlap to some extent. In describing the content characteristics of the tweet or video post, we considered the descriptive text, photo, and video included in the message. Manufacturers and influencers post videos in a variety of YouTube categories, including education, sports, entertainment, people and blogs, science and technology, and more.

**Table 4:** Characteristics of YouTube firearm influencers by total channel views.

Influencer channel	Subscribers	Total channel views	Description of influencer	Most viewed video
hickok45	4,201,360	1,143,690,107	Retired middle school teacher Greg Kinman collaborates with his son to review historic and modern firearms	460 Magnum versus Watermelons
DemolitionRanch	6,832,879	983,053,254	Popular YouTube personality and veterinarian Matt Carriker produces vlog-style gun reviews	How Deep into Dirt Will It Go?
FPSRussia	6,569,240	851,074,661	Now inactive; was one of the first and most popular gun channels on YouTube	AA-12 Fully Automatic Shotgun!!!
Iraqveteran8888	2,168,324	561,054,559	Iraq War veteran Eric Blandford makes gun and podcast videos with strong second amendment themes	RANGE TEST: THE ULTIMATE AR-15 MALL NINJA TACTICAL ZOMBIE DESTROYER!
Active Self Protection	1,385,888	557,194,826	Navy Veteran John Correia posts videos of him analyzing various self-defense encounters taken from security camera videos	More Proof that Evil Exists in Our World | Active Self Protection
Edwin Sarkissian	1,874,572	428,975,314	Entrepreneur makes range shooting videos with his friend	How many PUBG Cast Iron skillets does it take to stop a bullet?
Forgotten Weapons	1,211,469	359,042,434	Ian McCollum showcases historical guns and modern guns with unique histories	World's Smallest Pistol—2.7mm Kolibri
FullMag	2,406,155	352,093,297	Richard Ryan and a team of others make gun videos featuring a related brand, Black Rifle Coffee, and military themes	Will Bulletproof Glass Stop A .50 Cal? slow motion Richardson Ryan
nutnfancy	796,686	298,717,269	A retired Air Force pilot reviews guns, knives and weapon accessories. His videos occasionally feature his son, photos of military service, and his wife	Weasel vs Ground Squirrel: Nature's Combat
sootch00	837,830	231,285,288	Sootch makes gun review and podcast style videos with his sons and daughter: “God bless America, long live the republic.”	Classic Firearms Tour! Surplus Gun Heaven!
TFB TV	652,752	175,649,925	A team of people produce high quantity of gun review videos especially at events like the SHOT Show	Top 5 Hilariously Bad Carry Guns | TFBTV
Military Arms Channel	946,843	158,139,701	Tim Harmsen, Marine Corps veteran, reviews modern and historical military guns; he is known for his anti-NRA^a^ stance	5.7×28mm versus 22 Magnum
Total	29,883,998	6,099,970,635	N/A^b^	N/A

^a^NRA: National Rifle Association.

^b^Not applicable.

**Table 5:** Characteristics assessed in Twitter and YouTube activity of major firearm manufacturers and firearm influencers.

Characteristics	Description
Handgun	Handgun or pistol present in photo, text, or video
Shotgun	Shotgun present in photo, text, or video
Rifle	Rifle present in photo, text, or video
Attributes	Characteristics of firearm described in photo, text, or video
Protection	Post indicates or exhibits firearm use for protection
Hunting	Post indicates or exhibits hunting themes
Recreation	Post indicates or exhibits firearm use for recreation
2A	Post indicates or references the US Constitution’s Second Amendment
NRA	Post references the National Rifle Association
Conceal Carry	Post indicates or references firearm for concealed carry
Family	Post indicates or exhibits family themes
Kids	Post indicates or exhibits child use of firearms
Female	Post includes a woman or quotes a woman
Patriotism	Post indicates or exhibits patriotic themes (flags, leaders, and American pride)
Veterans	Post references or exhibits veterans
Military	Post references or exhibits military themes (soldiers, military use of weapons, and endorsement)
Police	Post references or exhibits law enforcement themes (police, thin blue line, and endorsement)
Western	Post references or exhibits western or cowboy themes
Weblink	Post links to website with sales, visually displays the link, or presents the link verbally
Gun Brand is Mentioned^a^	Video or description identifies specific gun brand(s)
Gun-Related Paid Promotion^a^	Video or description features paid promotion from the firearm or ammunition company
Nongun-Related Paid Promotion^a^	Video or description features paid promotion from the nongun company

^a^Additional characteristics identified for influencers.

## Results

### Quantitative Aggregation

A quantitative aggregation of the themes found in social media advertisements made by gun manufacturers and influencers helps characterize the prevailing strategies used by companies to increase purchases of consumer guns. To summarize our findings in social media advertising, we compared the use of themes across social media platforms, and we explored the types of guns advertised, advertised uses of guns, and how women, military, and police themes enter into advertising.

### Content Density and Gun Types in Firearm Advertising

The content and themes presented in YouTube posts exceeded the information conveyed by Twitter posts. An average of 2.2 themes was found for our sample of Twitter posts (n=928); whereas, twice as many themes, 4.4, were found in YouTube posts by manufacturers (n=425), and 5.3 themes were found on average among YouTube influencer videos (n=239). In summary, we found that twice as many themes are communicated in YouTube posts, compared with Twitter posts, indicating a higher density of content per YouTube post.

We found that handguns were advertised more frequently by manufacturers, and rifles were advertised more frequently by YouTube influencers. In comparison to shotguns or rifles, manufacturers on Twitter and YouTube more frequently display or refer to handguns or pistols (311/928, 30.2% of Twitter posts, and 243/425, 56.1% of YouTube posts). YouTube influencers, however, displayed rifles more often than other gun types (134/239, 58.3% of posts displayed rifles). This means that over half of the YouTube posts made by manufacturers intend to promote handguns but over half of the YouTube influencers promote rifles.

### Gun Use in Advertisements

**Figure 1:**
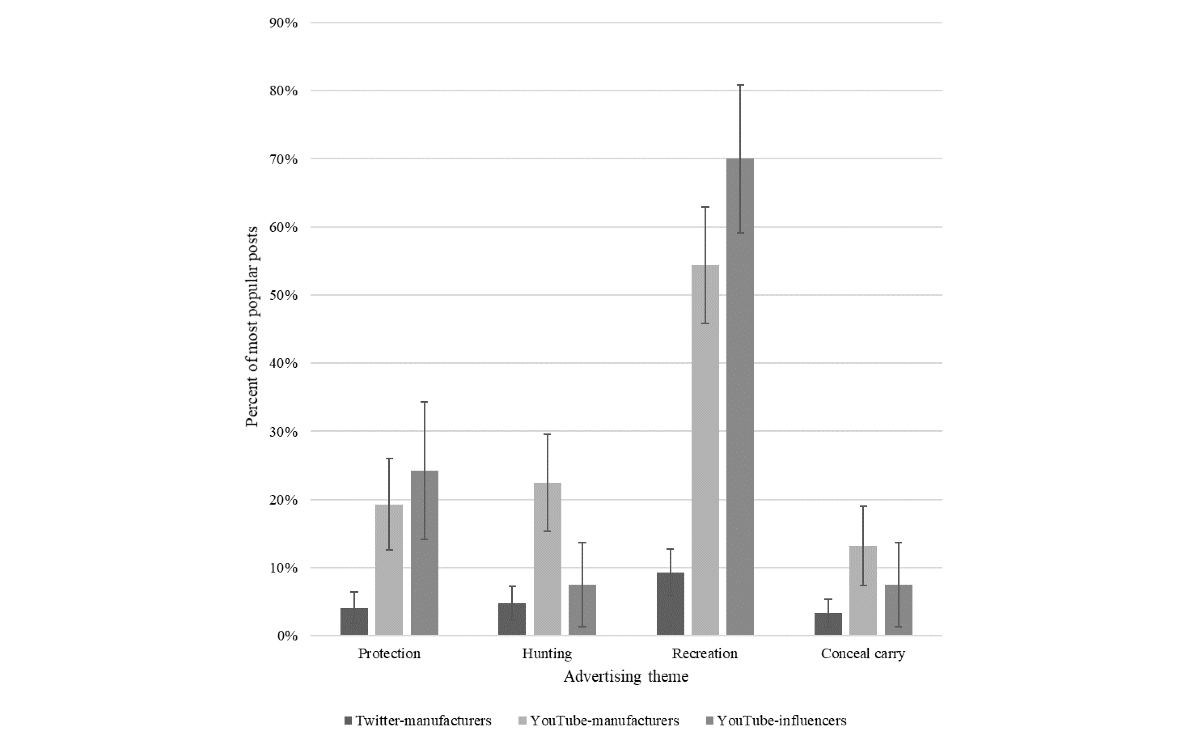
Suggested use for guns by most popular posts (1% CIs displayed).

Of the major themes we examined, recreation was among the most common themes in YouTube video posts. We identified the recreational use for posts that displayed or discussed gun range shooting or shooting targets. The hunting use was classified in its own category (see Figure 1 for comparison). When we split the sample to explore only the most viewed YouTube posts, we found that 54.4% (124/228) of posts by manufacturers and 70.0% (84/120) of influencer posts displayed recreational gun use.

Military, patriotic, and law enforcement themes were also commonplace: 46.7% (56/120) of the top viewed influencer posts depicted military themes. Retweets from all branches of the armed forces were identified among Twitter posts made by gun manufacturers. Patriotic theme prevalence varied depending on the source: 1 in 5 of the most retweeted Twitter posts (108 of 484 posts), 1 in 4 of the most viewed YouTube posts by manufacturers (63 of 228 posts), and 1 in 3 of the most viewed YouTube posts by influencers (40 of 120 posts) conveyed US patriotism.

### Women, Children, and Other Themes in Gun Advertising

**Figure 2:**
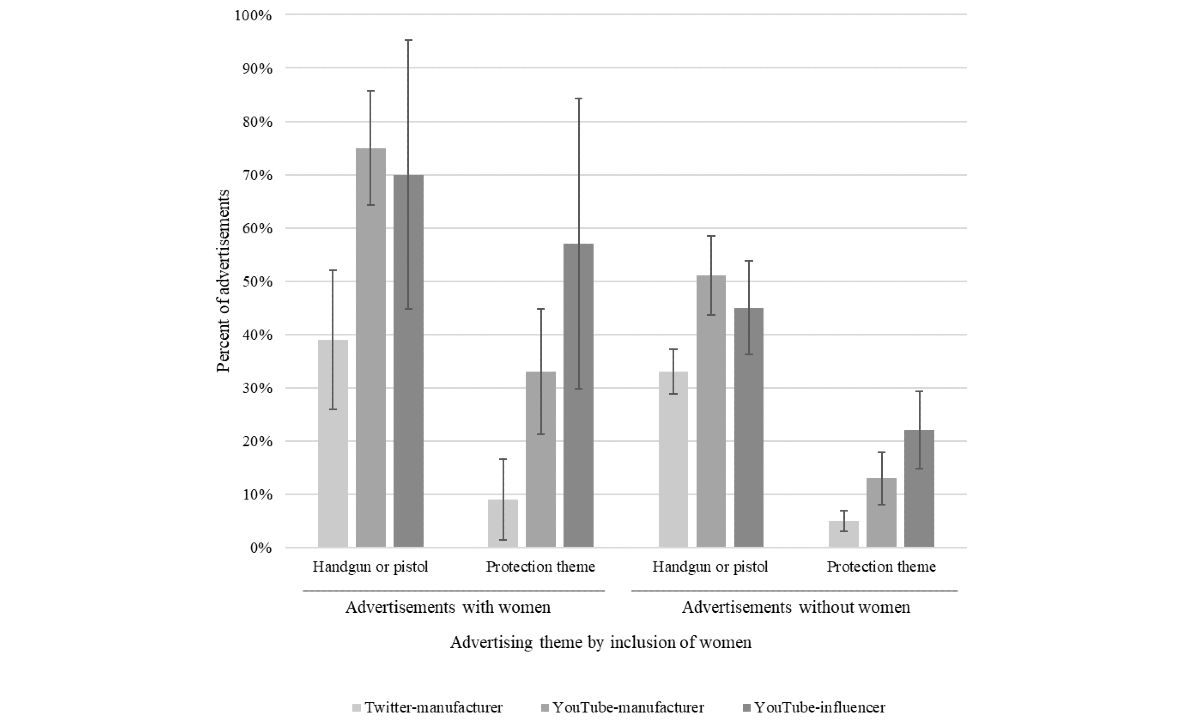
Prevalence of handgun- and protection-themed advertising in advertisements with and without women (1% CIs displayed).

The appearance of women in firearm advertising also varied significantly by source: the presence of a female in an advertisement was found in more than 1 out of 5 YouTube posts by manufacturers (108 of 425 posts), but in less than 1 out of 10 posts on Twitter (93 of 928 posts) or in those made by YouTube influencers (23 of 239 posts). Of the posts that do include women, handguns and gun protection themes were more prevalent (Figure 2).

Family themes were found in fewer than 10% of Twitter or YouTube posts. In all, 4.0% (17/426) of YouTube posts by manufacturers included children. Posts with children showed young children observing or participating in gun fire at shooting ranges, displaying hunting weapons, or receiving firearms as gifts. Although posts with children are less common than the other themes explored, children appear twice as often in video posts with women. The once common western and cowboy themes were found less than 5% of the time across sources.

Second amendment themes were also found in less than 5% of YouTube posts and 6.8% (64/928) of Twitter posts. The National Rifle Association (NRA) mentions or promotions were identified in 9.1% (85/928) of Twitter posts, 2.1% (9/425) of YouTube manufacturing posts, and 14.6% (35/239) of YouTube influencer posts. Although we also coded a theme labeled *attribute*, we found that the vast majority of gun advertisements describe the attributes of the guns. We chose to exclude this category from substantive comparison.

### Promotion of Internet Sales

**Figure 3:**
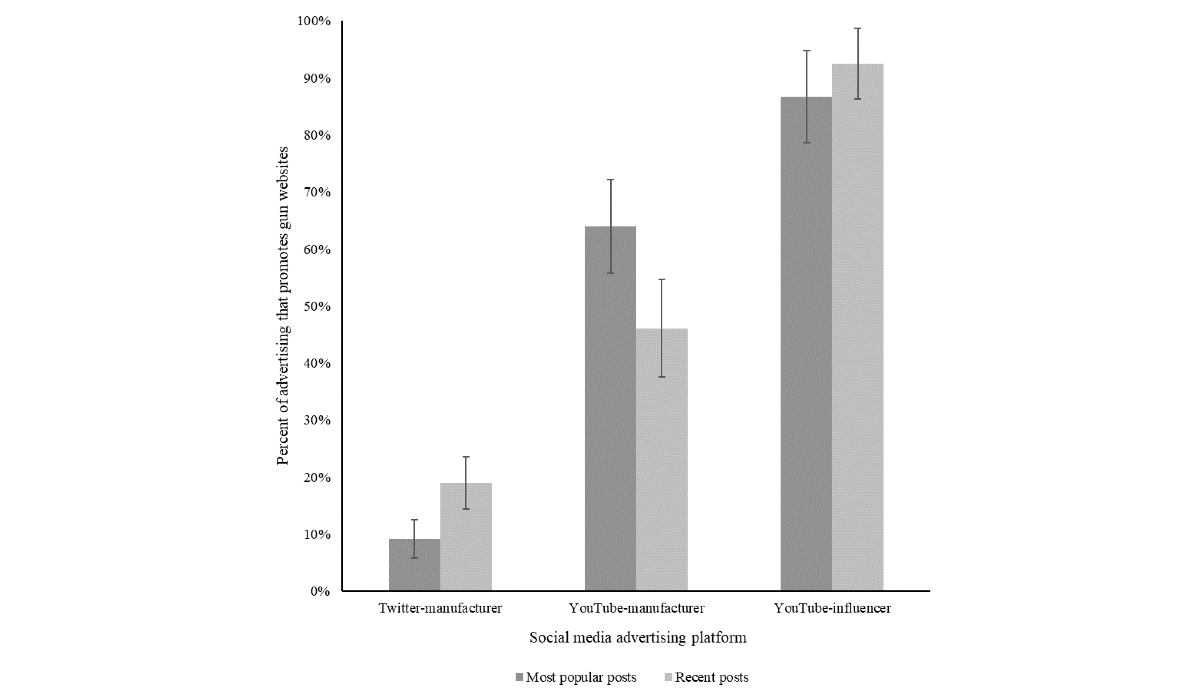
Percentage of recent and most popular posts that promote gun sales websites by social media type (1% CIs displayed).

In contrast to generally stated social media policies, social media posts connect viewers to websites with gun sales (Figure 3). YouTube posts more frequently provided links than Twitter posts. YouTube influencer posts were the most likely to link to gun sales: 8 out of 10 of the most popular YouTube influencer videos connected viewers to sales (104 of 120 posts), and 9 out of 10 of the most recent videos connected viewers to sales (111 of 120 posts).

In total, 64.4% (154/239) of YouTube influencer posts mention specific gun brand names, and 19% (45/239) include gun-related paid promotions. In total, 6.6% (16/239) of YouTube influencer posts contain nongun paid promotions; 18.9% (91/480) of recent Twitter posts made by gun manufacturers link to websites, but only 9.0% (44/484) of the most popular Twitter posts link to websites. Half of the YouTube videos by manufacturers provided links to internet sales (228 of 425 posts).

Among Twitter posts, firearm giveaways were not uncommon, and some manufacturers advertise and giveaway *builds* for custom rifles. At the time of analysis, firearm sponsorship in social media depicted gun use by the Major League Baseball pitcher, Andrew Cashner; company logos on a National Hockey League zamboni for the Nashville Predators; and as a featured sponsor of the National Association for Stock Car Auto Racing. Actress Halle Berry and actor Keanu Reeves were featured in video and photo Twitter posts for Sig Sauer MPX. One of the most popular retweeted posts across all manufacturers was a post by Anderson Rifles, who retweeted a post originally made by Texas governor, Greg Abbott: “I’m EMBARRASSED: Texas #2 in nation for new gun purchases, behind CALIFORNIA. Let’s pick up the pace Texans. @NRA.” The post also linked to the *Houston Chronicle* article that reported the state ranks for new gun purchases [77].

### Examples of Gun Video Content

Exploitation of stranger rape and glorification of military gun use were easily found in contemporary gun advertising. The following descriptions of a few important case studies exhibit gun advertising themes of protection for women, sexualization of gun use, promotion of assault weapons use for nonmilitary buyers, and arms production for the purpose of undermining gun control. The guns featured in these examples include Glock handguns, assault rifles and pistols from Sig Sauer, and AK-47s from Palmetto State Armory.

The *GLOCK and Gunny—Wrong Girl* advertisement was first posted by Glock on YouTube in 2013. This 2-min video is the most viewed manufacturer video with over 1.3 million views. In the video, a young woman at home, watching television in her pajama shorts and top, is being stalked by a bearded man in a van. She hears a knock at the door, does not see anyone, and goes to her room to remove a gun, presumably loaded, from a safe under her bed. Banging at the front door continues. She stands pointing her handgun at the door, and when it bursts open, the attacker faints. The police arrest him. No words are spoken until R Lee Ermey, playing the medic who constrains the attacker, concludes: “Somebody picked the wrong girl.” A link to the Glock website is shown across the screen.

The Glock video appears to be well received, with over 7000 likes, and a like to dislike ratio of 9:1. A user with the pseudonym *UziNineMillomeetah* commented, “Smokin’ hot red head who likes Glocks? My dream girl.” The video conveys handgun use for home protection and protection for women against stranger rape, while normalizing gun use and supporting defensive gun use as the first and only self-defense tactic. Gun use is sexualized. R Lee Ermey posted an *Extended Version* on his website, which received over 3 million views, but is identical to the post by Glock. Other videos in this series of advertisements include *GLOCK and Gunny—Wrong Convenience Store* and *Gunny & Glock—Wrong Guy*. All of the advertisements in this series convey the message that Glock guns are for protection: a message that also seeks to undercut public health evidence that the possession of firearms is a major risk factor for violent death from homicide and suicide and accidental death by a firearm.

Another category of advertisement worth exploring includes messages that glorify combat weaponry for private consumption. The Sig Sauer Sig MCX VIRTUS Mission series, including 4 advertising videos with a combined 0.75 million views, labeled under the YouTube category *Sports*, offers a case study in promotional militarism. The advertisements include the following: Mission 1: Overwatch, Mission 2: Target Identified, Mission 3: Vehicle Assault, and Mission 4: Tango Down. These videos feature gun use in cinematic combat settings, specifying the location as Ramadi, in Iraq. In *Mission 1*, the Sig MCX VIRTUS is shown in use by a sniper. The text on the Mission 1 post declares: “The world’s most innovative battle rifle, the SIG MCX VIRTUS, is ready for any mission.” The text on YouTube accompanying the Mission 2 video includes links to the sales website and explains, “Built from the ground up for suppressed operation, the MCX VIRTUS can go places - and do things - that no other rifle can.” Two messages are evident from these advertisements: first, the Sig MCX VIRTUS is intended for combat and lethality and second, Sig Sauer seeks to provide products with highly customized combat performance features to gun buyers.

Although Glock and Sig Sauer chose highly tailored video advertisements, Palmetto State Armory, a US gunmaker of AK-47s, posted a candid description of the company’s history and goals. Injured veteran and CEO Jamin McCallum stated the following:

I hope in thirty years people look back and say…we tried to pass gun control…but it wasn’t very effective…because this pesky company made 20 million ARs…they got them into circulation…and now the regulations we put into effect have little effect because there’s so much of it out there already.

Normalization of gun use with the intent to *reach the masses*, was an object of discussion by YouTube influencer, sootch00, who stated the following:

We have got to stay on YouTube because this is where the masses are coming. I’m telling you guys all over the place, people tell me all the time: “I got on YouTube, I saw a gun video, I was like, oh, I hadn't even thought about guns in a long time.” Before long they were watching different gun videos. They went out and bought a gun. They got to concealed carry. They joined the NRA, or GOA, or whatever. They became second amendment activists. If I go to Full30, I’m just preaching to the choir.

Full30 is a reference to a firearm video website that is sponsored by firearm manufacturers and contains almost exclusively videos on guns. Palmetto State Armory’s video collection includes a video of *Sarah*, a gunsmith, demonstrating how to assemble an AK-47 from parts that can be purchased from the manufacturer [78].

YouTube influencers occasionally give weapons poor reviews. In these moments, influencers may convey certain health warnings on a variety of factors. Influencers observed *gas in your face* from weapons use, commented on offending smells, eye irritation, potential risks of hearing loss, concerns about extremely high temperatures of the firearms after use, and observations about weight and recoil of weapons. Complaints like these by military personnel have been important in directing health research to explore the respiratory effects of occupational gunfire, including two recent Norwegian studies that found significant declines in forced expiratory volume even 24 hours after shooting practice, and reports of respiratory symptoms similar to metal fume fever in almost all participants [79,80]. The authors explain that “soldiers are exposed to emissions of CO, particulate matter (dust), combustion products, copper, zinc, bismuth, lead and tin,” and “bullets without potentially harmful emissions are not available” [80]. Unfortunately, these important health messages are lost to the more common tropes from influencers describing the *fun* of recreational shooting.

When Saylor et al [10] explored firearm advertising in gun magazines from 2001 to 2002, they found a circulation of 4.2 million, with hunting and outdoors (20.4%) and patriotism (15.0%) as the most common themes identified in their sample. These themes continue to be prevalent. However, the reach of videos, based on measurements of views, appears to be significantly wider than print materials.

## Discussion

### Principal Findings

We demonstrate that firearm manufacturers use Twitter and YouTube to promote the sale of guns to millions of viewers. The renewed interest in and attention to firearm advertising permits an important deepening and sophistication to public debate in the United States on the scope of possible responses to gun violence. Like all consumer products, firearms have a life cycle that requires scrutiny and is subject to intervention through multiple phases: design, manufacturing, retail, distribution, and disposal.

A focus on firearm advertising demonstrates that marketing to promote retail and distribution is one area, among many, that may be considered for both research and revised rule making. Better understanding the life cycle of guns raises both opportunities and significant challenges to where and how we work as communities to reduce lethal violence. This section first explores opportunities for changes in the US legislation that can help reduce the widespread proliferation of small arms in the United States by limiting advertising and suggests avenues for future research. After identifying opportunities, major challenges are listed to propose subsequent research and dialog on these more embedded obstacles to achieving peaceful communities.

### Opportunities

Specific changes in the legislation are possible. The American Public Health Association collaborated with 14 partners to write to the US Congress in favor of federal research funding on gun violence, currently limited by the 1996 Dickey amendment [81]. Several public health scholars have argued in favor of repealing the PLCAA [14,40]. Repealing the PLCAA would remove protections for manufacturers and dealers from lawsuits related to harmful gun use and false advertising pertaining to gun use as a form of protection.

Organizations such as Moms Demand Action have made progress in lobbying retail stores, such as Starbucks and Target, to prohibit guns from their premises. Furthermore, private sector policies may shape retail behavior. The *Washington Post* reported an example where the software marketing agency, Salesforce, threatened to withhold services for distributors of military-style rifles [82]. Both private and public actions, from grassroots and from the national stage, can help build a culture of nonviolence; and, should the PLCAA and Tiahrt Amendment be repealed, 1 advertisement agency has already proposed graphic warning labels for ammunition [83].

The work presented here studies how firearm manufacturers use social media for advertising. However, present advocacy efforts to reduce gun violence have made significant progress in connecting gun control advocates by using social media to promote community engagement and campaigns to incentivize corporate changes. For example, the Moms Demand Action campaign explicitly discusses the strategy to use social media to unite gun control advocates [84]. Every town for Gun Safety [85] has similarly adopted a social media approach. Web-based media coverage of gun violence from *The Trace* provides an example of generating a counternarrative to the NRA’s Web-based media [86]. Academic consortiums, such as those emerging from work by the Coalition to Stop Gun Violence, can assist particularly during strategic opportunities when lawmakers are focused on gun violence [87]. All this is to say gun control advocacy and its relative success in relation to gun promotion deserves deeper consideration and can contribute to best practice guidelines or *lessons learned* materials for local health departments and advocacy groups.

Additional research can contribute to our understanding of firearm advertising. For instance, future work in this area could explore the local use of social media advertising by small business producers of firearms and the interaction of these uses with larger companies. The state of New Jersey offers a simple example. Among the states with relatively few firearm manufacturers, New Jersey ranked 46th in the number of firearm manufacturers, with 28 federally licensed firearm manufacturers and one major producer: Henry Repeating Arms. However, 79% (22/28) of New Jersey manufacturers maintain websites, most of which connect users to sales and social media. Although the sales may be relatively small for these producers, their community impact on firearm social media may be significant.

As suggested in the literature on health communications generally [88], understanding the media commonly viewed by gun owners can help in the design of brochures, posters, webpages, and informational materials that may reinforce recommendations made by physicians on the topic of gun ownership (eg, facts on gun ownership and health risks introduced into homes with guns and evidence of decades of predatory advertising for the purpose of increasing sales). Twitter is also used by local health departments for health communications, and scholars have suggested that these programs might engage community members, to a greater extent, in a dialog about local health [89]. Social media also offers the possibility for public health engagement in the chat features, as were identified in some protobacco YouTube posts [52]. Some research has gone as far to suggest that policy might help ensure a prohealth balance to available media [90].

### Obstacles

The globalization of media presents an obstacle for regulation, although some countries do legislate restrictions [91]. An emerging concern from the public health literature on social media and tobacco use is that social media are not neutral platforms. Social media companies are commercial entities, not public goods, despite efforts by these companies to appear free and open [71,72]. Significant progress made toward restrictions on tobacco, eg, are facing reversal owing to the combination of e-cigarettes, internet advertising, and internet sales. Youths’ use of e-cigarettes has been declared an epidemic [92].

The power of industry, where full-time employees are paid to produce advertising content—photographs, videos, entertainment, and compelling messaging—for social media platforms, is not counteracted by advocacy messaging or ordinary people who hold the majority opinion [93]: firearm ownership and tobacco use are not health behaviors that should be encouraged or modeled. Firearm manufacturers are making efforts to fully exploit social media channels for commercial purposes.

The incentive to profit from consumer products exceeds the current social capacity to counterpose these messages, suggesting that health interventions are necessary. However, guidelines are available for communicating public health objectives effectively [94,95]; and, several organizational structures serve to advocate for changes in corporate behavior: national organizations, coalitions, health professionals and researchers, legal groups, local organizations, and other participants in campaigns [37]. These groups can, then, engage in information gathering, legislative action, electoral activities, litigation, actions aimed at corporations, and education, information, and mobilization campaigns [37]. Roberts suggests that democratic policy interventions and partnerships with public libraries may be more suitable approaches to combating misinformation spread by social media than working within social media settings [72]. This paper contributes to the larger discussion about opportunities for regulating the media that promote harmful health behaviors in the United States and internationally.

Unfortunately, rather than entering at the peak of adoption, public health work is now addressing firearms, tobacco, and marijuana in a phase of escalation. From this point of view, gun violence and health effects from tobacco, e-cigarettes, and marijuana are anticipated to increase and not decline in the foreseeable future. As some firearm manufacturers and dealers have increased sales of gun parts to avoid regulation, concerns about circulation and social media distribution of 3D printed guns remain on the horizon. A future legislation and policy intervention to limit the advertising and content viewed by children is likely [70]. Such work might consider the characteristics and strategies used by influencers.

The domestic production and distribution of small arms in the United States contributes to regional and global health challenges. While reporting on the trial of Joaquin Guzman, the Mexican drug lord known as *El Chapo*, the PBS Newshour stated that “one of El Chapo’s deputies testified that 99 percent of the guns he purchased came from the United States” [96]. Actions taken to understand and intervene in the life cycle of US firearms can reduce gun violence not only in the United States, but regionally and around the world. In this way, by focusing upstream on gun production, US public health actors can make substantive contributions to the UN Sustainable Development Goal 16: “Promote peaceful and inclusive societies for sustainable development, provide access to justice for all and build effective, accountable and inclusive institutions at all levels.” Specifically, Target 16.1 seeks to “significantly reduce all forms of violence and related death rates everywhere” [97].

In building consensus and shifting cultural norms on gun ownership and use, US military and law enforcement present the largest obstacles, but also the most important opportunities for reducing gun violence. Public health collaboration with the military and law enforcement is absolutely necessary for building effective actions. For example, public health review, health impact assessments, and environmental impact assessments of military and law enforcement small arms purchasing and communications could provide agencies with vital information about the civilian and regional health consequences of their choices.

Any restrictions on military and law enforcement agencies from training soldiers and police officers on gun safety in the home should be lifted. Section 1062 of Public Law 111-383, which “forbids the Department of Defense to ‘collect or record’ any information about private firearms of members of the military or its civilian employees,…” [14] should be repealed. Social media produced by the military and law enforcement currently works to the benefit of firearm producers: future social media produced by these government agencies should consider opportunities to promote peace and stability, in our homes, communities, and around the world. Perhaps the Department of Defense (DoD) might reconsider social media use altogether given the commercial, nonpublic, and obscurely regulated nature of these platforms.

In some cases, helpful changes to firearm advertising could emerge from enforcement of existing policy. According to DoD instructions on visual information:

Service members must comply with DoD 5500.07-R, DoDI 8550.01, and DoDI 1334.01 prior to permitting NFEs (Non-Federal Entities) to use their image in uniform. Both active duty Military Service members and former members are prohibited from wearing their uniform in connection with commercial interests when an inference of official sponsorship for the activity or interest could be drawn. [98]

Several influencers, such as nutnfancy, may be in violation of this policy, and the DoD may be able to take steps to reduce the reach of influencers who gain credibility in promoting firearm sales by visually displaying their current or past military experience. The DoD disclaimer, “The appearance of U.S. Department of Defense (DoD) visual information does not imply or constitute DoD endorsement,” was not identified in firearm advertising that used armed service symbols and images. Sig Sauer, eg, explicitly uses DoD visual material as an endorsement, without disclaimers.

### Limitations and Future Research

By focusing primarily on social media advertising, we were not able to demonstrate why, despite tighter restrictions on cable advertising, firearm production rose and stayed high, during both Obama and Trump administrations. A market analysis that includes careful consideration of the firearm economy over the past decade may help reveal the causes in the overall purchasing trends for guns. Twitter and YouTube may be used for a time-series analysis and possibly for uncovering the relative market power of social media in influencing global firearm sales.

The methodology that this paper pursued was based on previous research on print advertising. By selecting the most recent and most popular Twitter and YouTube posts, we generated a systematic search and analysis of major gun manufacturer and popular influencer use of these services. Our search was not a simple random sample. We cannot make inferences about the less-viewed influencers that we did not assess. We also cannot infer social media themes used by very small gun manufacturers. We were also not able to examine gun use on Instagram or Facebook, which are also commonly used social media services. The exploration of these forums is an opportunity for future research.

By choosing to evaluate the 20 most recent tweets and 10 most recent videos, the periods for the evaluated posts varied depending on the frequency and temporal distribution of posts made by manufacturers and influencers. We sought to explore variations in posts based on popularity and recency to identify differences between the two. Our intention was to characterize the advertising themes and presence of links to sales found in social media by top gun producers and influencers, and not to examine causal relationships. Future research could benefit from better understanding contemporary firearm consumers and the influence of advertising on their decision making.

Continued work in the domain of firearm advertising can help deepen the understanding of other themes and strategies used by manufacturers. For example, safety can be a specific theme explored in detail. Connections to religion were observed and worth exploring further. Race, ethnicity, and sexual orientation were not explored in detail here, but would be interesting to study further. Our work was limited to English-only social media: advertising in other languages would make for an interesting comparison. Podcasts are another new source of internet content distribution that would be helpful to analyze.

Social media and print media across audiences, specifically the police and military, need more attention. Gun advertisements appear to be printed by *staff writers* across magazines and Web-based sources. While researching for this paper, we found a Glock advertisement surface word-for-word in articles, not as advertisements or sponsored content, but as reader content for *Police* magazine [99] and *Military Times* [100]. The copied text includes links to the Glock website. How frequently are gun advertisements appearing as news articles? What are the perceptions about the legitimacy of this media? Who are the audiences for these magazines? Future research could help shed light on these questions.

The informational material on the US Army Picatinny Arsenal website, which engages in small arms acquisition for the armed forces, offers an interesting contrast to the way firearm advertisers characterize guns. The website describes the purpose of the arsenal as providing nearly 90% of the Army’s *lethality* [101]. Lethality seems a more suitable term for describing the purpose of firearms. In analyzing firearm advertising, we observed numerous instances of sharing of information from the armed services and promotional videos highlighting the bravery of specific servicemen. Future work could explore social media use by the US armed services and law enforcement to understand how these agencies respond to or incorporate advertising from firearm manufacturers into their information sharing with the communities that follow them.

## Abbreviations

ATF: Bureau of Alcohol, Firearms, Tobacco, and Explosives
DCM: Danger Close Media
DHSI: Digital Humanities Summer Institute
NRA: National Rifle Association
PLCAA: Protection of Lawful Commerce in Arms Act
